# New Azulene-Type Sesquiterpenoids from the Fruiting Bodies of *Lactarius deliciosus*

**DOI:** 10.1007/s13659-017-0130-1

**Published:** 2017-05-11

**Authors:** Michel Feussi Tala, Jianchun Qin, Joseph T. Ndongo, Hartmut Laatsch

**Affiliations:** 1 Institute of Organic and Biomolecular Chemistry, University of Goettingen, Tammannstrasse 2, 37077 Göttingen, Germany; 2 School of Plant Science, Jilin University, Xian Road No. 5333, Changchun, 130062 Jilin People’s Republic of China

**Keywords:** *Lactarius deliciosus*, Fungal pigments, Azulene sesquiterpenoids, Antibacterial activity

## Abstract

**Abstract:**

In the ^1^H NMR-guided fractionation of extracts from the edible mushroom *Lactarius deliciosus*, two new azulene-type sesquiterpenoids, 7-isopropenyl-4-methyl-azulene-1-carboxylic acid (**1**) and 15-hydroxy-3,6-dihydrolactarazulene (**2**), together with seven known compounds were characterized. Their structures were determined on basis of spectroscopic evidence, as well as by comparing with literature data. Amongst the known metabolites, the ^13^C NMR assignment of 15-hydroxy-6,7-dihydrolactarazulene (**3**) is reported here for the first time. Moreover, 7-acetyl-4-methylazulene-1-carbaldehyde (**5**) displayed a moderate antibacterial activity against *Staphylococcus aureus*.

**Graphical Abstract:**

*Digital image of *L. deliciosus.* Retrieved March 17, 2017 from https://upload.wikimedia.org/wikipedia/commons/e/e3/Lactarius_deliciosus_1_(1).jpg 
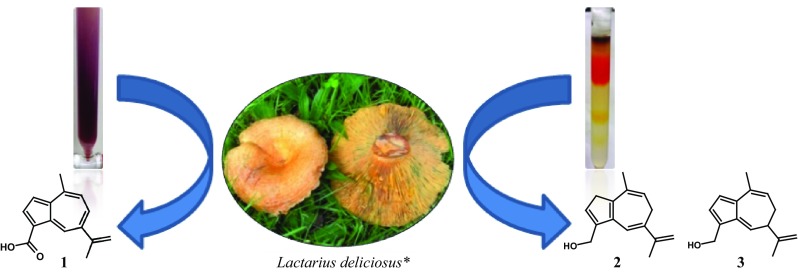
.

**Electronic supplementary material:**

The online version of this article (doi:10.1007/s13659-017-0130-1) contains supplementary material, which is available to authorized users.

## Introduction

The genus *Lactarius* belongs to the family Russulaceae (class Basidiomycetes) and is widely distributed allover the world. Many *Lactarius* species are edible; chemically, these mushrooms are appreciated for their metabolites, including sesquiterpenes, steroids, nitrogen-containing compounds and other secondary metabolites with e.g. antitumor or antiviral activities [1, 2]. *Lactarius deliciosus*, *L*. *aureus, L. hatsudake,* and others are well known for their colorful pigments, some of which are formed as response to injury of the fruiting bodies [3–5]. Previous chemical and biological investigation of this genus showed that these pigments belong to the group of azulene-type sesquiterpenoids, some of them possessing potent biological activities [6–10]. This class of metabolites is also considered as chemotaxonomic marker of the genus *Lactarius* [11].

As part of our search for fungal metabolites, we are reporting herein the structures and complete ^1^H and ^13^C NMR assignments of two new pigments **1** and **2** from the edible mushroom *L. deliciosus* collected in the forests near Göttingen (Germany). In addition to these two new compounds, four other pigments (**3**–**6**) and three fatty acids were also isolated. Although ^1^H NMR data of the unstable dihydroazulene alcohol **3** have been reported [3, 12], to the best of our knowledge, no ^13^C NMR data were published for this compound. We also evaluated the antimicrobial activities of the pigments **1** and **4**–**6**.

## Results and Discussion

**Fig. 1:**
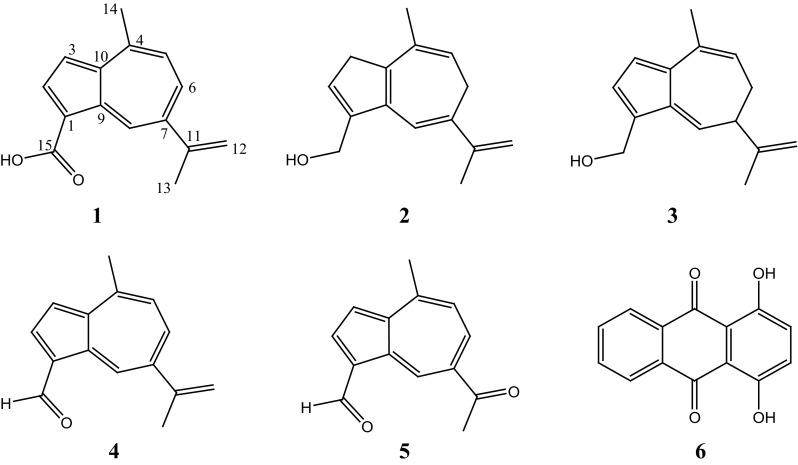
Structures of pigments **1**–**6** isolated from the fruiting bodies of *Lactarius deliciosus*

**Fig. 2:**
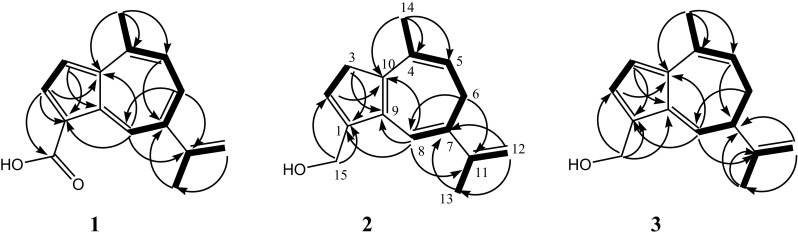
Key HMBC (*arrows*) and COSY (*bold bonds*) correlations for compounds **1**–**3**

After extraction of the freshly collected mushrooms with methanol and repetitive chromatography on Sephadex LH-20, compound **1** was obtained as a purple amorphous solid, with violet color in solution. Electrospray high resolution mass spectrometry (ESI HRMS) displayed an [M + H]^+^ ion peak at *m/z* 227.1075, suggesting C_15_H_14_O_2_ as molecular formula. The ^1^H and ^13^C NMR data of **1** (Table 1) were very similar to those reported for lactaroviolin (**4**) [13], a further *Lactarius* constituent also isolated in the present investigation. The ^1^H–^1^H COSY spectrum of **1** exhibited three spin systems of H-2/H-3, H-5/H-6/H-8/H-14 and H-12/H_2_-13, indicating that **1** and **4** might have the same azulene substitution pattern. Interestingly, the ^1^H NMR spectrum (Table 1) of **1** displayed a proton at *δ*_H_ 10.0 (d, *J* = 2.1 Hz), which was attached to a carbon at *δ*_C_ 136.8 (HSQC), excluding thereby an aldehyde. This finding corroborated with the ^13^C NMR spectrum (Table 1) that did not show resonances at lower field of an aldehyde group, but exhibited the characteristic signal of a conjugated carboxyl group at *δ*_C_ 170.0. Further comparison of ^1^H and ^13^C NMR data of **1** with those of lactaroviolin (**4**) and 7-acetyl-4-methylazulene-1carboxylic acid [13] were in good agreement with the presence of the carboxy group at C-1. This was supported in the HMBC experiment by long-range couplings between the aromatic proton at *δ*_H_ 8.43 (H-2) and the carboxy group (Fig. 2). The HMBC spectrum also exhibited cross-peaks of the olefinic methylene protons at *δ*_H_ 5.46 and 5.33 (H_2_-12) with the carbon atoms at *δ*_C_ 23.3 (C-13), 146.9 (C-11) and 141.2 (C-7), and of the methyl protons at *δ*_H_ 2.96 (H-14) with the carbons at *δ*_C_ 143.3 (C-10), 148.0 (C-4) and 129.9 (C-5). From the above data, the structure of **1** was established as 7-isopropenyl-4-methyl-azulene-1-carboxylic acid (Fig. 1). Related azulene-1-carboxylic acids have been characterized previously from *L. deliciosus* [13] and *L. hadsudake* [8].

**Table 1:** ^1^H NMR and ^13^C NMR data of compounds **1**–**3** (*δ* in ppm, *J* in MHz)

No.	**1** ^a^		**2** ^b^		**3** ^b^	
	*δ*_H_	*δ*_C_	*δ*_H_	*δ*_C_	*δ*_H_	*δ*_C_
1	–	116.5	–	147.4	–	139.1
2	8.43 (d, 4.3)	140.6	6.23 (br s)	125.6	6.24 (brs)	127.3
3	7.30 (d, 4.3)	115.3	3.20 (br s)	40.4	6.33 (brs)	129.0
4	–	148.0	–	132.7	–	132.1
5	7.44 (d, 10.8)	129.9	5.13 (td, 7.1, 1.5)	115.8	5.50 (br t, 7.1)	121.8
6	7.88 (dd, 10, 2.1)	136.2	2.48 (m)	28.5	2.46 (m)	30.8
					2.26 (ddd, 15.4, 7.1, 3.1)	
7	–	141.2	–	130.8	3.23 (m)	44.8
8	10.00 (d, 2.1)	136.8	6.49 (br s)	117.4	6.77 (d, 4.5)	143.0
9	–	140.2	–	141.8	–	142.4
10	–	143.3	–	145.7	–	135.2
11	–	146.9	–	141.7	–	146.4
12	5.46 (s)	116.3	5.42 (s)	114.0	4.81 (m)	111.5
	5.33 (s)		5.08 (s)		4.75 (m)	
13	2.36 (s)	23.3	1.96 (s)	21.1	1.77 (s)	20.9
14	2.96 (s)	25.0	1.93 (s)	20.5	1.88 (s)	21.9
15	–	170.0^c^	4.33 (br s)	58.1	4.31 (br s)	56.6

^a^ In CDCl_3_

^b^ In DMSO

^c^ From HMBC data

Further compounds were isolated as inseparable orange pigments, which decomposed rapidly. ESI MS exhibited *pseudo*-molecular ion peaks at *m/z* 237 [M + Na]^+^, 253 [M + K]^+^, and 467 [2M + K]^+^, consistent with the molecular formula C_15_H_18_O. The ^1^H and ^13^C NMR spectra indicated, however, that it was a mixture of two isomeric compounds **2** and **3** in a ratio of 1:3. The ^1^H and ^13^C NMR spectra of compounds **2** and **3** showed significant differences to those of **1** and **4**, notably the signals of two aliphatic methylene groups in **2** and one methylene and one methine group in **3**. Carbonyl signals were absent, and the methyl signal of C-14 at the azulene system was shifted upfield. The ^1^H NMR spectra of both compounds indicated two additional oxymethylene protons, which were connected with *sp* ^3^ carbons at *δ*_C_ 58.1 and 56.6, respectively, indicating the presence of the dihydroazulene alcohols **2** and **3**; the latter is known to be very unstable [3, 6, 12].

We were unable to separate both compounds, however, succeeded to assign all NMR signals in the mixture and elucidated the structures of **2** and **3** unambiguously; the ^1^H–^1^H COSY spectrum was clearly resolved and indicated three spin systems for each component (see Fig. 2). The different signal intensities in the 1:3 mixture of **2** and **3** allowed us to assign the HSQC and HMBC signals without doubt. For the less intensive peaks (compound **2**), the methylene protons at *δ*_H_ 4.33 (H_2_-15) correlated with the carbons at *δ*_C_ 147.4 (C-1) and 125.6 (C-2); the expected correlation with C-9 was observed for compound **3**, but not for **2** (Fig. 2). These two carbons in turn correlated with the methylene protons at *δ*_H_ 3.20 (H_2_-3). HMBC correlation was also observed between C-1 and the olefinic proton at *δ*_H_ 6.49 (H-8), which showed further correlations with the carbons at *δ*_C_ 28.5 (C-6), 141.7 (C-11), and 145.7 (C-10). A long-range correlation was seen from the methyl at *δ*_H_ 1.93 (H-14) to C-10, and to the carbons at *δ*_C_ 132.7 (C-4) and 115.8 (C-5). Thereby, the minor component was finally characterized as 15-hydroxy-3,6-dihydrolactarazulene (**2**).

In a similar way, the main component was identified as 15-hydroxy-6,7-dihydrolactarazulene (**3**). Its structure was further supported by comparison of the ^1^H NMR data with those published by Sterner et al. [3]. To the best of our knowledge, the ^13^C NMR data (Table 1) for this compound are reported here for the first time.

Further components of the extract were identified on basis of their NMR data as lactaroviolin (**4**) [13, 14], 7-acetyl-4-methylazulene-1-carbaldehyde (**5**) [13], quinizarin (**6**) [15], stearic acid, and a mixture of oleic and linoleic acid. Among them, quinizarin (**6**) is reported here from higher fungi for the first time.

Compounds **1** and **4**–**6** were screened for their antimicrobial activities against *Bacillus subtilis*, *Staphylococcus aureus*, *Escherichia coli*, *Candida albicans* and *Mucor miehei*. Only 7-acetyl-4-methylazulene-1-carbaldehyde (**5**) displayed a moderate antibacterial activity against *S. aureus* in the agar diffusion test, with an inhibition diameter of 15 mm at a concentration of 50 μg/disc.

## Experimental Section

### General Experimental Procedures

The NMR spectra were recorded on a Varian Inova-500 spectrometer at 599.737 MHz (^1^H) or 150.818 MHz (^13^C), respectively. The chemical shifts are given in *δ* values with TMS as internal reference, and coupling constants are given in [Hz]. The ESI and ESI HR mass spectra were recorded on a Bruker micrOTOF mass spectrometer. Open column chromatography was done on silica gel 60 (0.063–0.20 mm), and PTLC was performed on silica gel P/UV_254_ (both obtained from Macherey–Nagel, Düren, Germany). Size exclusion chromatography was performed on Sephadex LH-20 (Lipophilic Sephadex; Amersham Biosciences, Ltd., purchased from Sigma-Aldrich Chemie, Steinheim, Germany). Pre-coated silica gel plates (Polygram SIL G/UV_254_, Macherey–Nagel & Co.) were used for TLC. Spots were visualized at 254 or 365 nm, and sprayed with an anisaldehyde/sulfuric acid reagent followed by heating.

### Extraction and Isolation

The fruiting bodies of *L. deliciosus* were collected in forests near Göttingen (Germany) in September 2015. The fresh material (7.8 kg) was grinded and macerated with 2 × 4 L of MeOH at ~20 °C. The solution was evaporated under reduced pressure to afford a dark brown crude extract (110 g) that was suspended in water and further extracted with ethyl acetate (EtOAc). The evaporation residue (38 g) of the EtOAc fraction was chromatographed on Sephadex LH-20 (column 7 × 60 cm) with MeOH to give four main fractions A–D. According to their ^1^H NMR profiles, the colorless fractions A (8.5 g) and B (6.3 g) contained fatty acids and glycerol derivatives, while the orange and violet fractions C (11.7 g) and D (10.5 g), respectively, contained azulene-type sesquiterpenoids. Fraction C was again chromatographed on Sephadex LH-20 (column 4 × 90 cm) and further on silica gel (column, CH_2_Cl_2_) to afford a mixture of the unstable compounds **2** and **3** (35 mg). Fraction D crystallized from MeOH to yield stearic acid (200 mg). The mother liquor from D was purified again on Sephadex LH-20 (column, CH_2_Cl_2_/MeOH 1:1) to afford compound **1** (5 mg), quinizarine (**6**; 2 mg, *R*_f_ = 0.90 CH_2_Cl_2_/MeOH 95:5) and two subfractions D-1 (violet) and D-2 (red). By PTLC (CH_2_Cl_2_/MeOH 99:1), D-1 gave lactaroviolin (**4**; 70 mg, *R*_f_ = 0.78 CH_2_Cl_2_/MeOH 95:5). Purification of D-2 on a silica gel (column, CH_2_Cl_2_/MeOH 98:2) yielded 7-acetyl-4-methylazulene-1-carbaldehyde (**5**; 7 mg, *R*_f_ = 0.72 CH_2_Cl_2_/MeOH 95:5) and a mixture of stearic, oleic and linoleic acid (150 mg).

### 7-Isopropenyl-4-methyl-azulene-1-carboxylic acid (**1**)

Purple amorphous solid, *R*_f_ = 0.41 (CH_2_Cl_2_/MeOH 95:5); UV (MeOH) *λ*_max_ (log ε) 238 (4.10), 300 (4.10), 380 (3.59); IR (film) ν_max_ 3300-2500 (br, OH), 2920, 1652 (C=O), 1496, 1415, 1252, 890 cm^−1^; ^1^H NMR (CDCl_3_, 600 MHz) and ^13^C NMR (CDCl_3_,125 MHz) data, see Table 1; (+)-ESI MS *m/z* 227 ([M + H]^+^), 249 ([M + Na]^+^), 475 ([2M + Na]^+^); (+)-ESI HRMS; *m/z* 227.1075 (calcd for C_15_H_15_O_2_ [M + H]^+^, 227.1067).

### 15-Hydroxy-3,6-dihydrolactarazulene (**2**) and 15-hydroxy-6,7-dihydrolactarazulene (**3**)

Orange gum, *R*_f_ = 0.30 (CH_2_Cl_2_/MeOH 95:5); ^1^H NMR (DMSO-*d*_6_, 300 MHz) and ^13^C NMR (DMSO-*d*_6_, 125 MHz) data, see Table 1; (+)-ESI MS: *m/z* = 237 ([M + Na]^+^), 253 ([M + K]^+^), 475 ([2M + K]^+^); (+)-ESI HRMS; *m/z* 237.1250 (calcd for C_15_H_18_ONa [M + Na]^+^, 237.1255).

### Antimicrobial Test

The antimicrobial test was performed according to a previously described procedure [16].

## Electronic supplementary material

Below is the link to the electronic supplementary material.

Supplementary material 1 (DOC 7066 kb)
