# Supplementary material for: New Azulene-Type Sesquiterpenoids from the Fruiting Bodies of Lactarius deliciosus
Source: Nat Prod Bioprospect. 2017 May 11;7(3):269–73. doi: 10.1007/s13659-017-0130-1 (PMC5481275; doi:10.1007/s13659-017-0130-1)
Supplement: Supplementary file 1 — Supplementary material 1 (DOC 7066 kb) [file 13659_2017_130_MOESM1_ESM.doc]

# Supporting Information

**New Azulene-Type Sesquiterpenoids from the Fruiting Bodies of *Lactarius deliciosus***

**Michel Feussi Tala, Jianchun Qin[[1]](#footnote-2), Joseph T. Ndongo, Hartmut Laatsch[[2]](#footnote-3)***

University of Goettingen, Institute of Organic and Biomolecular Chemistry, Tammannstrasse 2, D-37077 Goettingen, Germany

**Table of Contents**

F**igure S1:** Structures of pigments **1**-**6** isolated from the fruiting bodies of *Lactarius deliciosus* 4

**Figure S2:** (+)-ESI mass spectrum of 7-isopropenyl-4-methyl-azulene-1-carboxylic acid (**1**) 5

**Figure S3:** (**−**)-ESI mass spectrum of 7-isopropenyl-4-methyl-azulene-1-carboxylic acid (**1**) 6

**Figure S4:** ^1^H NMR spectrum (600 MHz, CDCl_3_) of 7-isopropenyl-4-methyl-azulene-1-carboxylic acid (**1**) 7

**Figure S5:** ^13^C NMR spectrum (125 MHz, CDCl_3_) of 7-isopropenyl-4-methyl-azulene-1-carboxylic acid (**1**) 8

**Figure S6:** HSQC spectrum (600 MHz, CDCl_3_) of 7-isopropenyl-4-methyl-azulene-1-carboxylic acid (**1**) 9

**Figure S7:** HMBC spectrum (600 MHz, CDCl_3_) of 7-isopropenyl-4-methyl-azulene-1-carboxylic acid (**1**) 10

**Figure S8:** COSY spectrum (600 MHz, CDCl_3_) of 7-isopropenyl-4-methyl-azulene-1-carboxylic acid (**1**) 11

**Figure S9:** (+)-ESI mass spectrum of 15-hydroxy-3,6-dihydrolactarazulene (**2**) and 15-hydroxy-6,7-dihydrolactarazulene (**3**) 12

**Figure S10:** ^1^H NMR spectrum (600 MHz, DMSO-*d*_6_) of 15-hydroxy-3,6-dihydrolactar­azulene (**2**) and 15-hydroxy-6,7-dihydrolactarazulene (**3**) 13

**Figure S11:** ^13^C NMR spectrum (125 MHz, DMSO-*d*_6_) of 15-hydroxy-3,6-dihydrolactarazulene (**2**) and 15-hydroxy-6,7-dihydrolactarazulene (**3**) 14

**Figure S12:** HSQC spectrum (300 MHz, DMSO-*d*_6_) of 15-hydroxy-3,6-dihydrolactar­azulene (**2**) and 15-hydroxy-6,7-dihydrolactarazulene (**3**) 15

**Figure S13:** Magnified HSQC spectrum (300 MHz, DMSO-*d*_6_) of 15-hydroxy-3,6-dihydrolactarazulene (2) and 15-hydroxy-6,7-dihydrolactarazulene (**3**) 16

**Figure S14:** Magnified HSQC spectrum (300 MHz, DMSO-*d*_6_) of 15-hydroxy-3,6-dihydrolactarazulene (**2**) and 15-hydroxy-6,7-dihydrolactarazulene (**3**) 17

**Figure S15:** Magnified HSQC spectrum (300 MHz, DMSO-*d*_6_) of 15-hydroxy-3,6-dihydrolactarazulene (**2**) and 15-hydroxy-6,7-dihydrolactarazulene (**3**) 18

**Figure S16:** HMBC spectrum (300 MHz, DMSO-*d*_6_) of 15-hydroxy-3,6-dihydrolactar­azulene (**2**) and 15-hydroxy-6,7-dihydrolactarazulene (**3**) 19

**Figure S17:** Magnified HMBC spectrum (300 MHz, DMSO-*d*_6_) of 15-hydroxy-3,6-dihydrolactarazulene (**2**) and 15-hydroxy-6,7-dihydrolactarazulene (**3**) 20

**Figure S18:** Magnified HMBC spectrum (300 MHz, DMSO-*d*_6_) of 15-hydroxy-3,6-dihydrolactarazulene (**2**) and 15-hydroxy-6,7-dihydrolactarazulene (**3**) 21

**Figure S19:** Magnified HMBC spectrum (300 MHz, DMSO-*d*_6_) of 15-hydroxy-3,6-dihydrolactarazulene (**2**) and 15-hydroxy-6,7-dihydrolactarazulene (**3**) 22

**Figure S20:** Magnified HMBC spectrum (300 MHz, DMSO-*d*_6_) of 15-hydroxy-3,6-dihydrolactarazulene (**2**) and 15-hydroxy-6,7-dihydrolactarazulene (**3**) 23

**Figure S21:** Magnified HMBC spectrum (300 MHz, DMSO-*d*_6_) of 15-hydroxy-3,6-dihydrolactarazulene (**2**) and 15-hydroxy-6,7-dihydrolactarazulene (**3**) 24

**Figure S22:** COSY spectrum (300 MHz, DMSO-*d*_6_) of 15-hydroxy-3,6-dihydrolactar­azulene (**2**) and 15-hydroxy-6,7-dihydrolactarazulene (**3**) 25

**Figure S23:** Magnified COSY spectrum (300 MHz, DMSO-*d*_6_) of 15-hydroxy-3,6-dihydrolactarazulene (**2**) and 15-hydroxy-6,7-dihydrolactarazulene (**3**) 26

**Figure S24: M**agnified COSY spectrum (300 MHz, DMSO-*d*_6_) of 15-hydroxy-3,6-dihydrolactarazulene (**2**) and 15-hydroxy-6,7-dihydrolactarazulene (**3**) 27

**1** **2** **3**

**4 5 6**

Figure S1: Structures of pigments 1-6 isolated from the fruiting bodies of *Lactarius deliciosus*


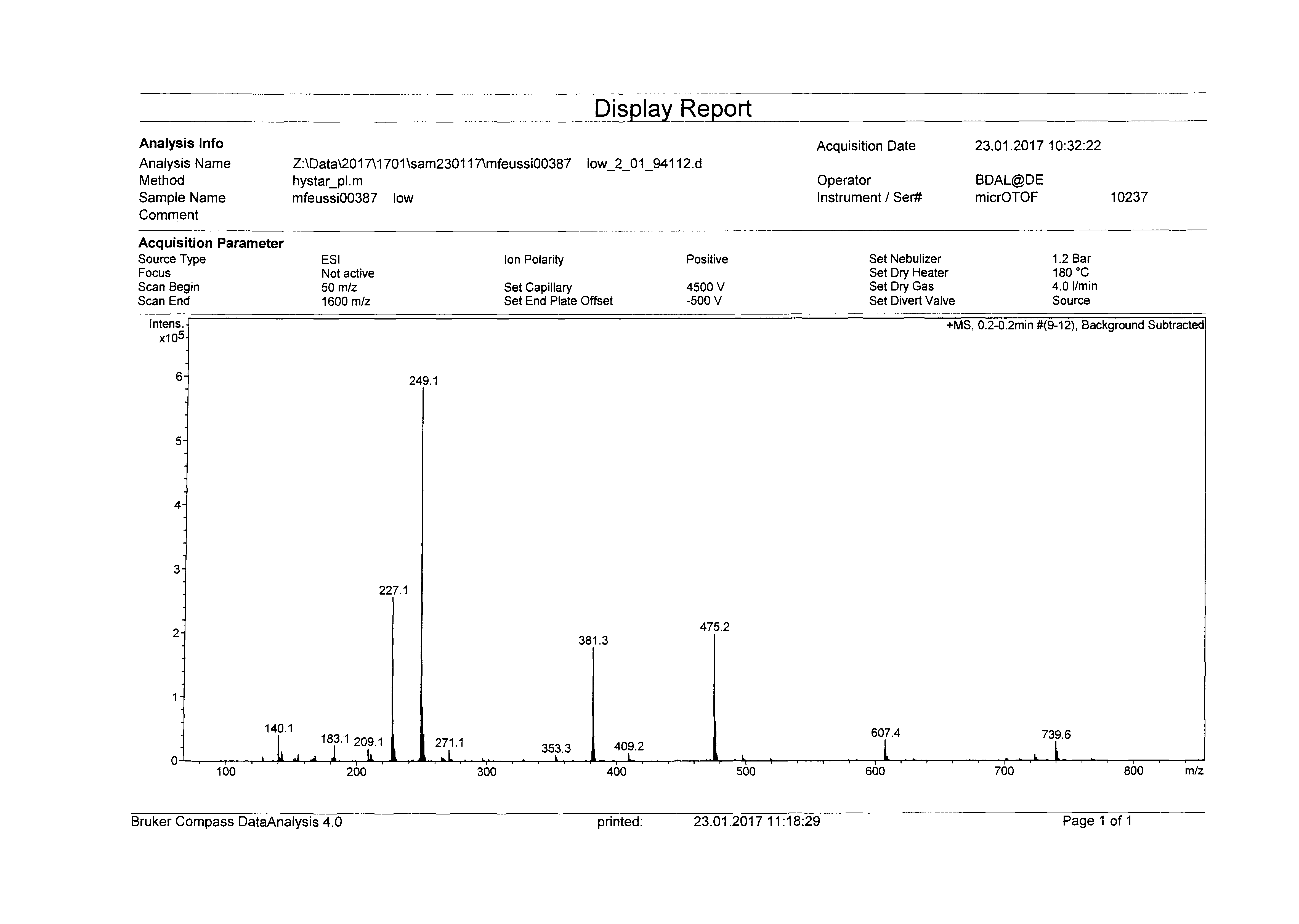


Figure S2: (+)-ESI mass spectrum of 7-isopropenyl-4-methyl-azulene-1-carboxylic acid (1)


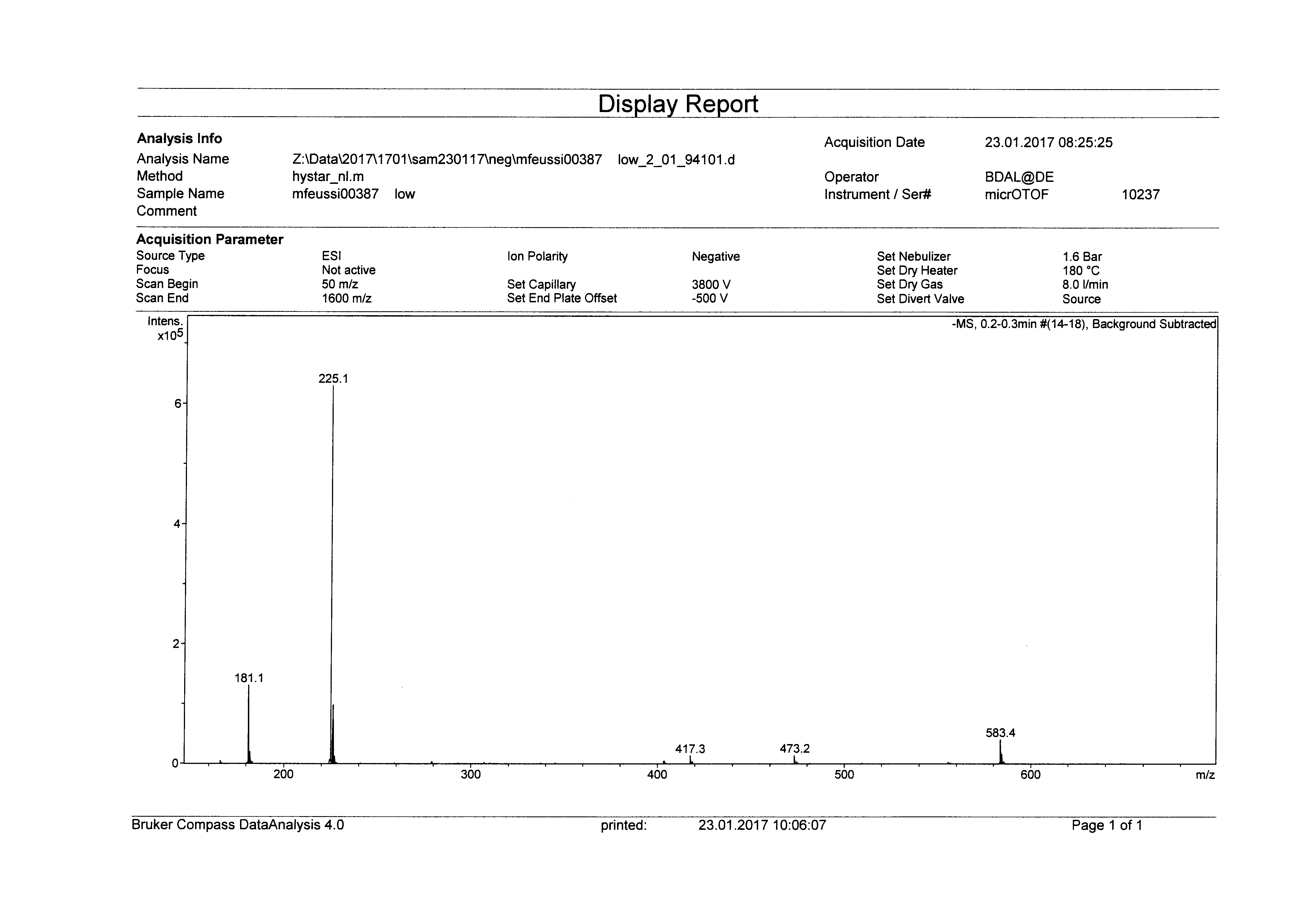


# Figure S3: (−)-ESI mass spectrum of 7-isopropenyl-4-methyl-azulene-1-carboxylic acid (1)

**
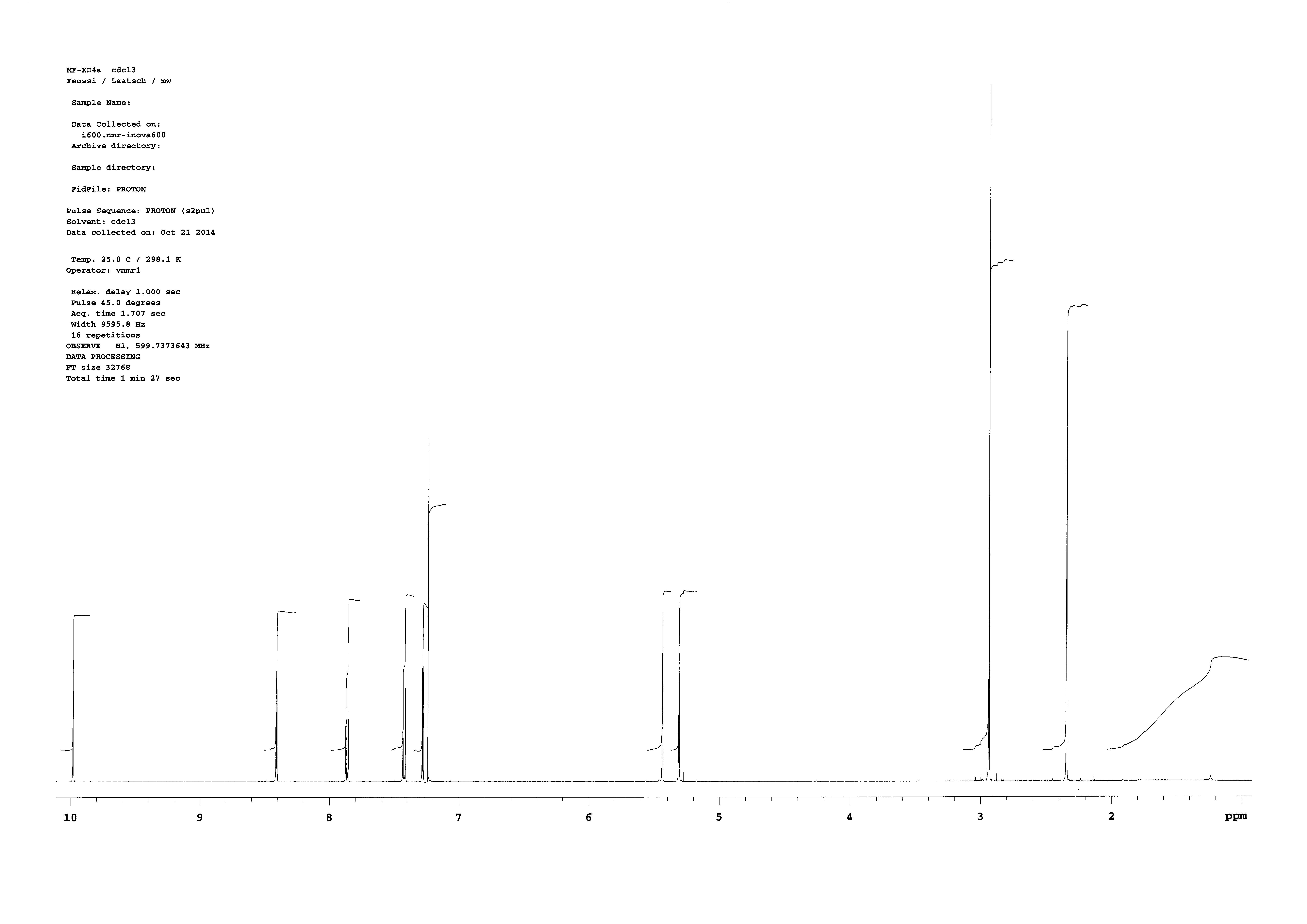
**

# Figure S4: ^1^H NMR spectrum (600 MHz, CDCl_3_) of 7-isopropenyl-4-methyl-azulene-1-carboxylic acid (1**)**


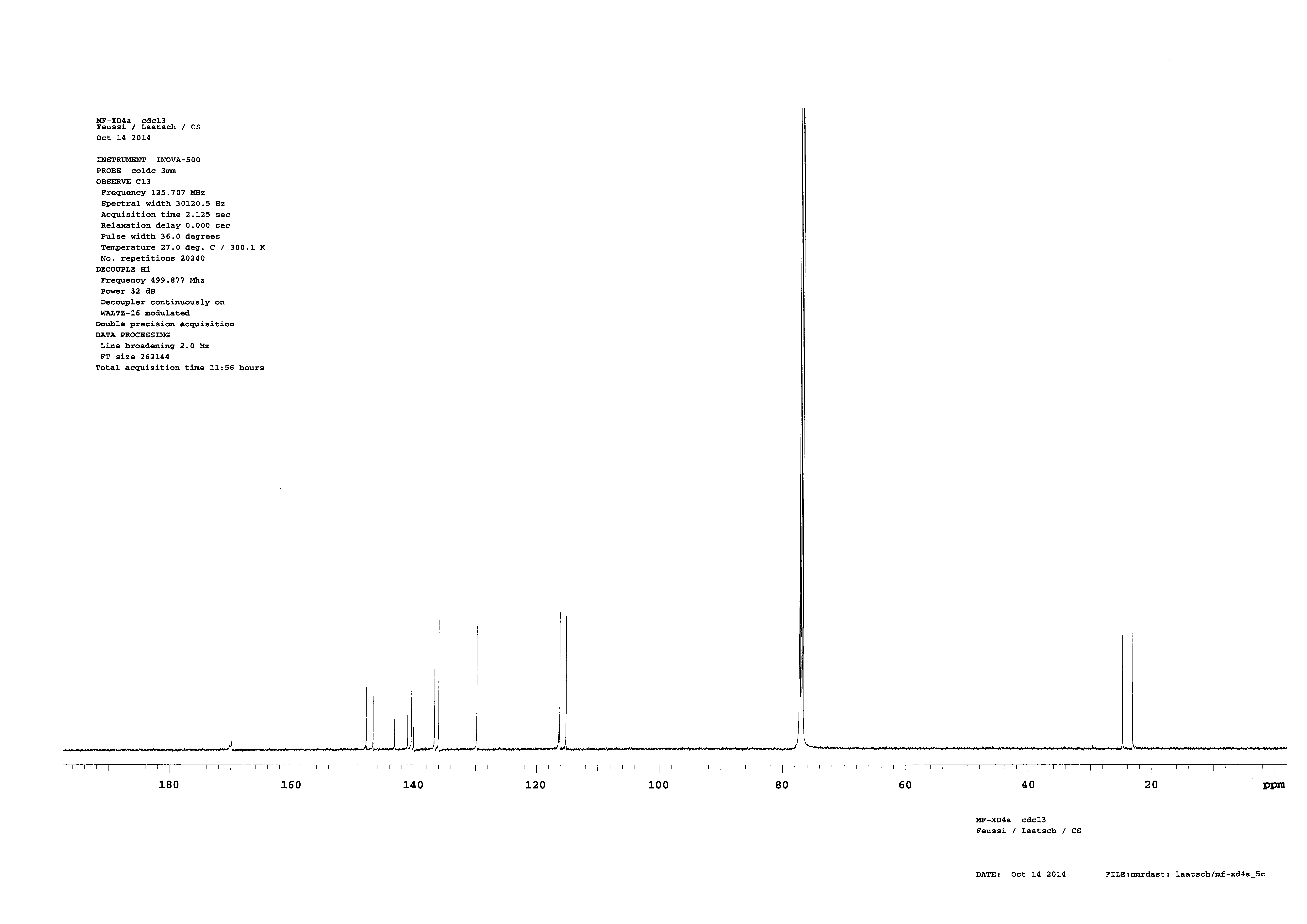


# Figure S5: ^13^C NMR spectrum (125 MHz, CDCl_3_) of 7-isopropenyl-4-methyl-azulene-1-carboxylic acid (1)


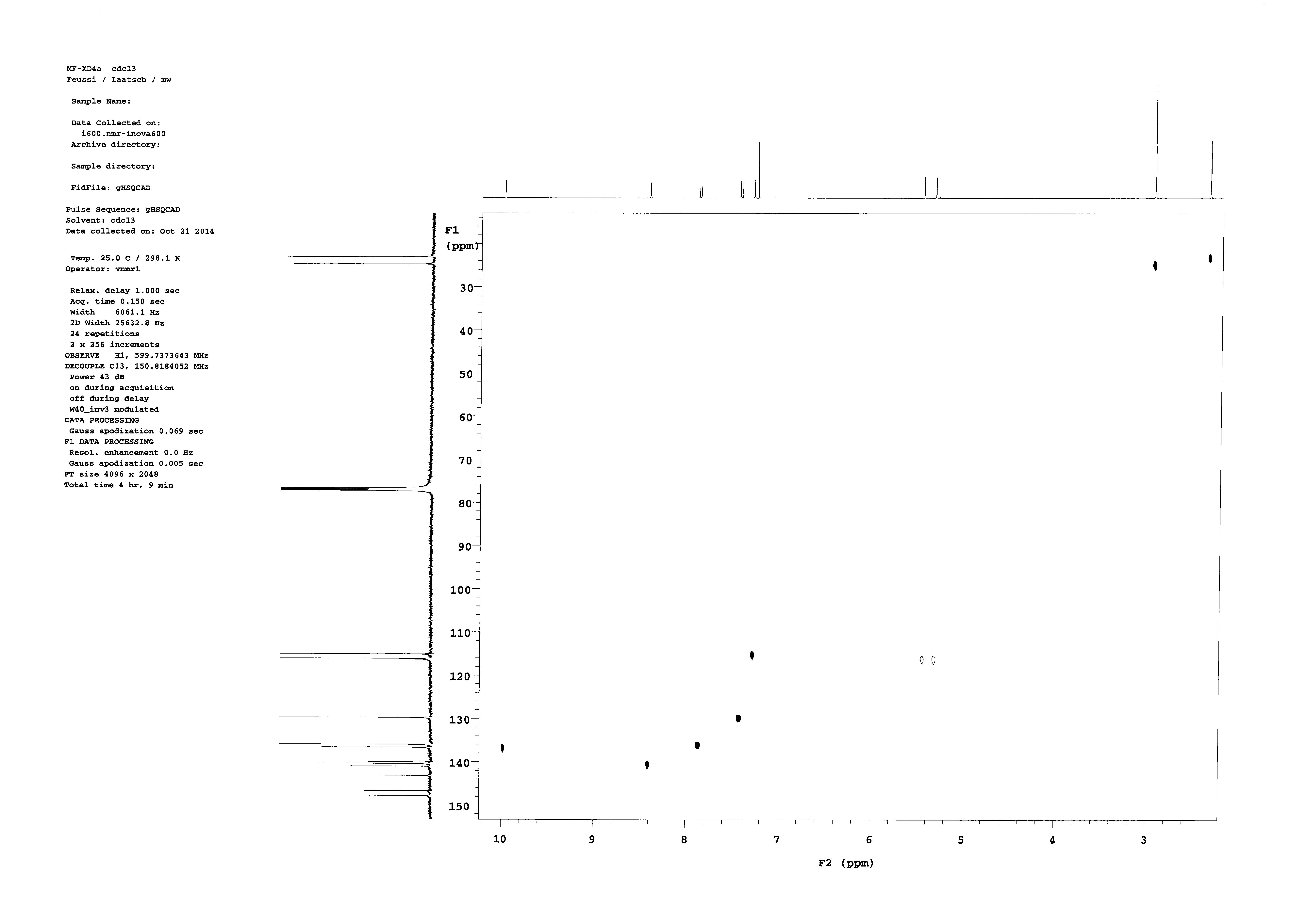


# Figure S6: HSQC spectrum (600 MHz, CDCl_3_) of 7-isopropenyl-4-methyl-azulene-1-carboxylic acid (1)


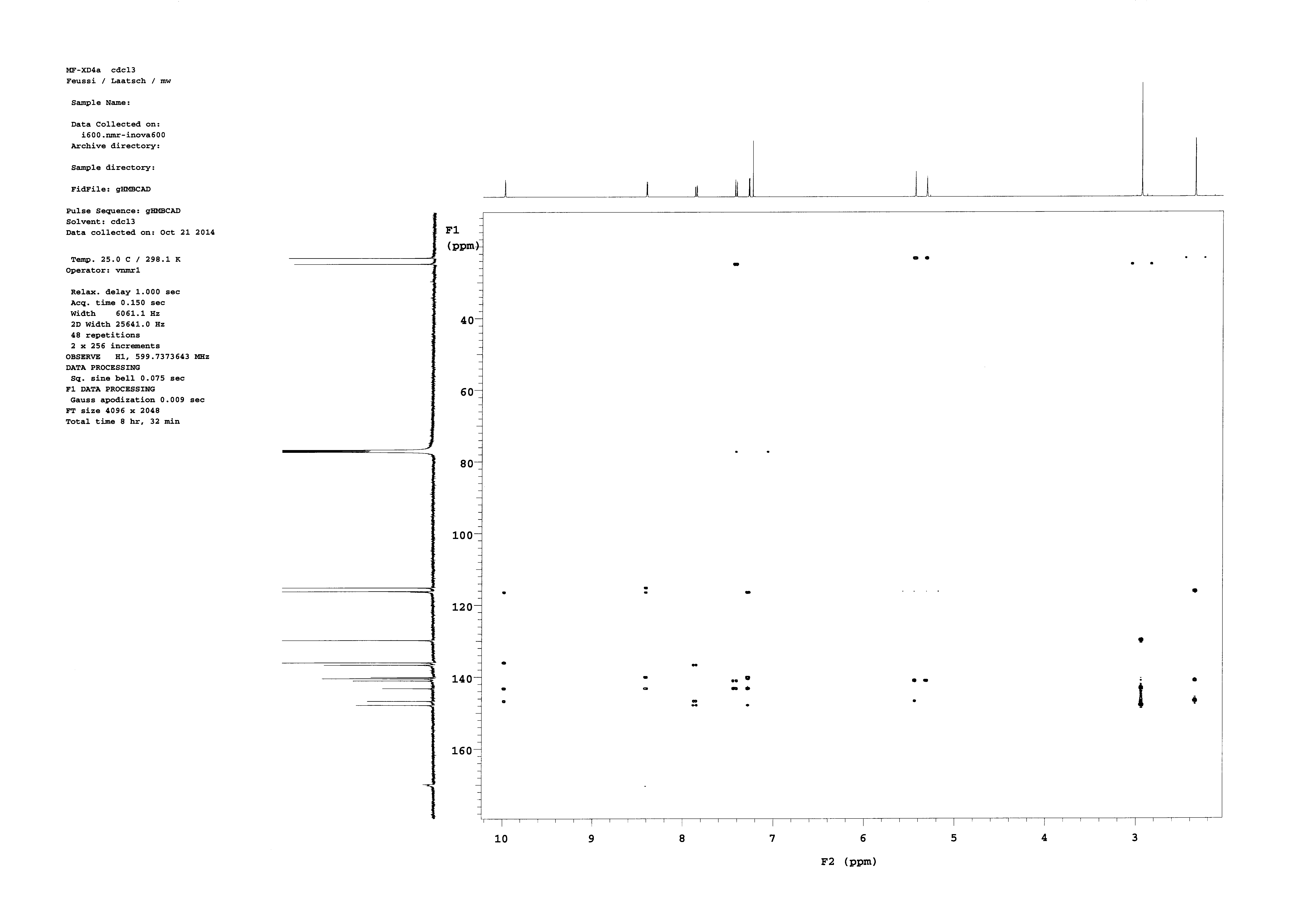


# Figure S7: HMBC spectrum (600 MHz, CDCl_3_) of 7-isopropenyl-4-methyl-azulene-1-carboxylic acid (1)


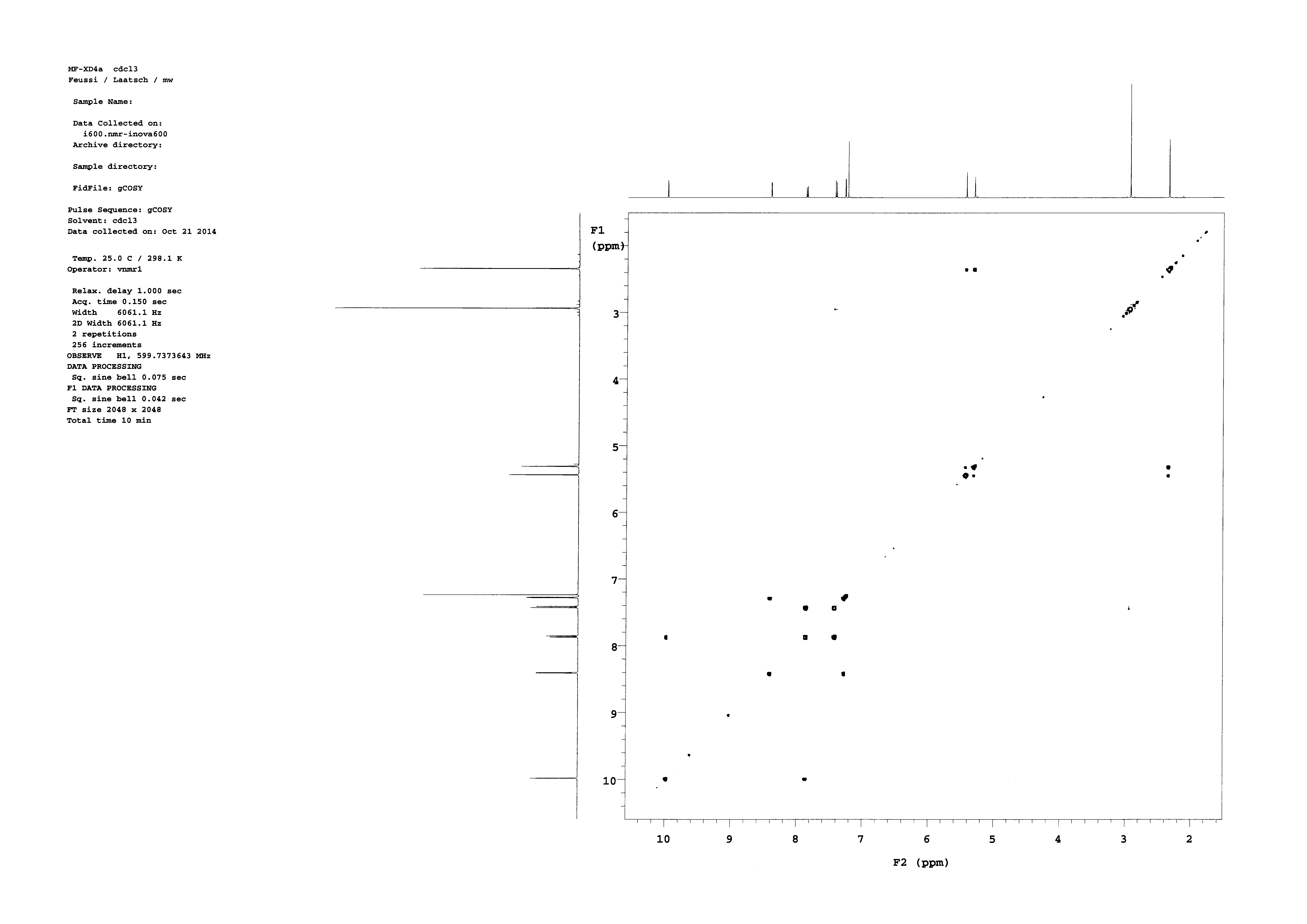


# Figure S8: COSY spectrum (600 MHz, CDCl_3_) of 7-isopropenyl-4-methyl-azulene-1-carboxylic acid (1)


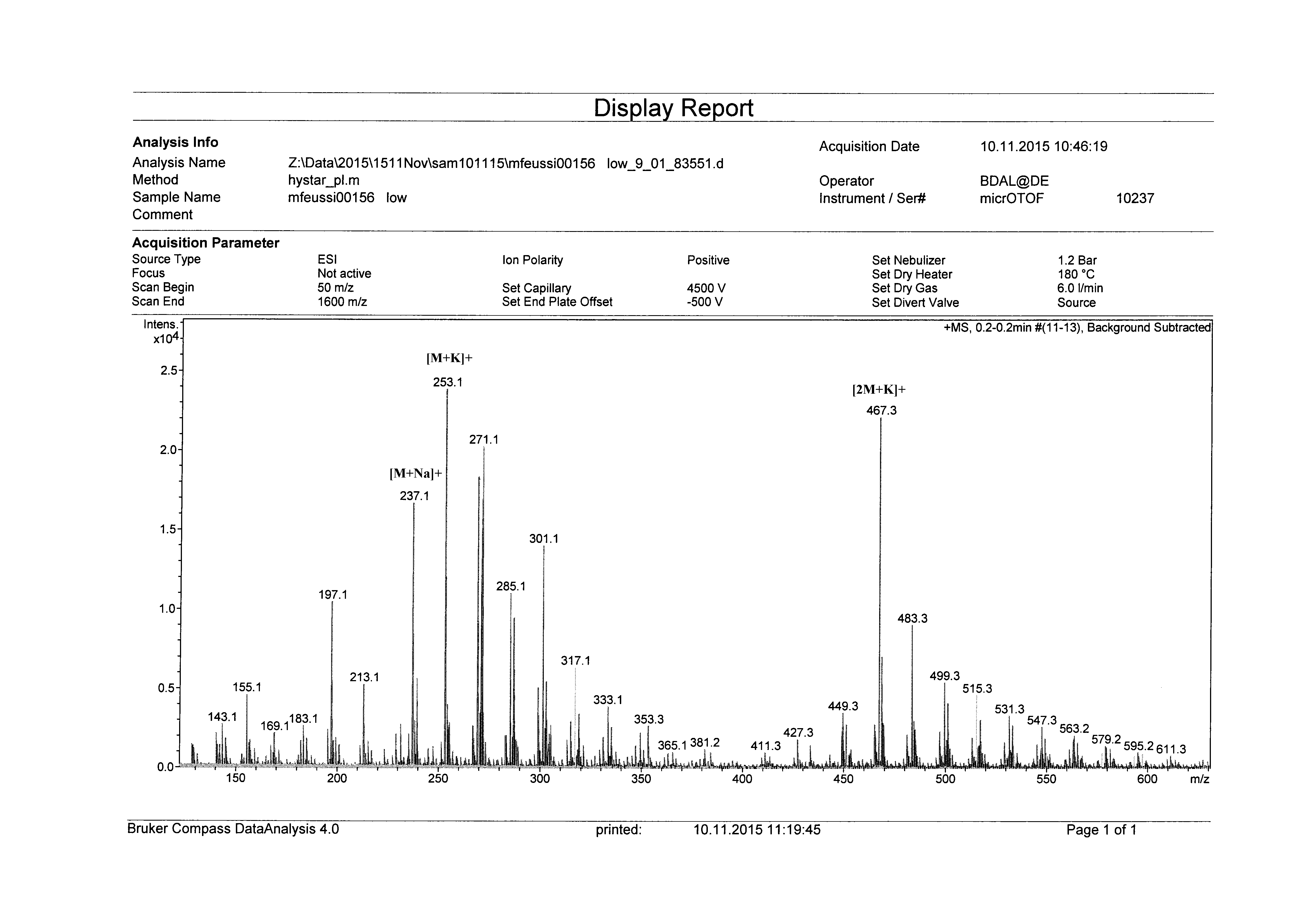


# Figure S9: (+)-ESI mass spectrum of 15-hydroxy-3,6-dihydrolactarazulene (2) and 15-hydroxy-6,7-dihydrolac­tarazulene (3)


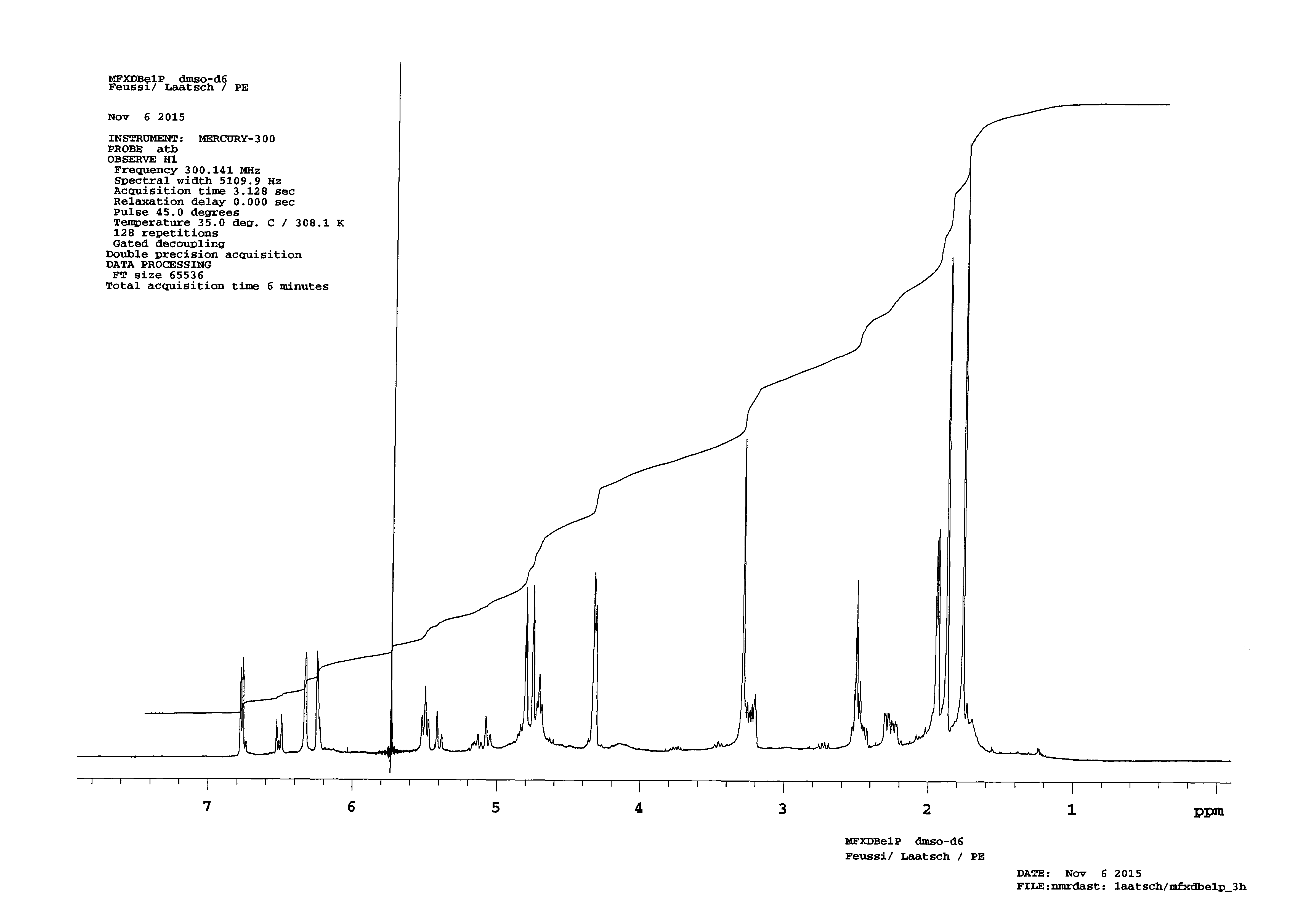


# Figure S10: ^1^H NMR spectrum (600 MHz, DMSO-*d*_6_) of 15-hydroxy-3,6-dihydrolactarazulene (2) and 15-hydroxy-6,7-dihydrolac­tarazulene (3)


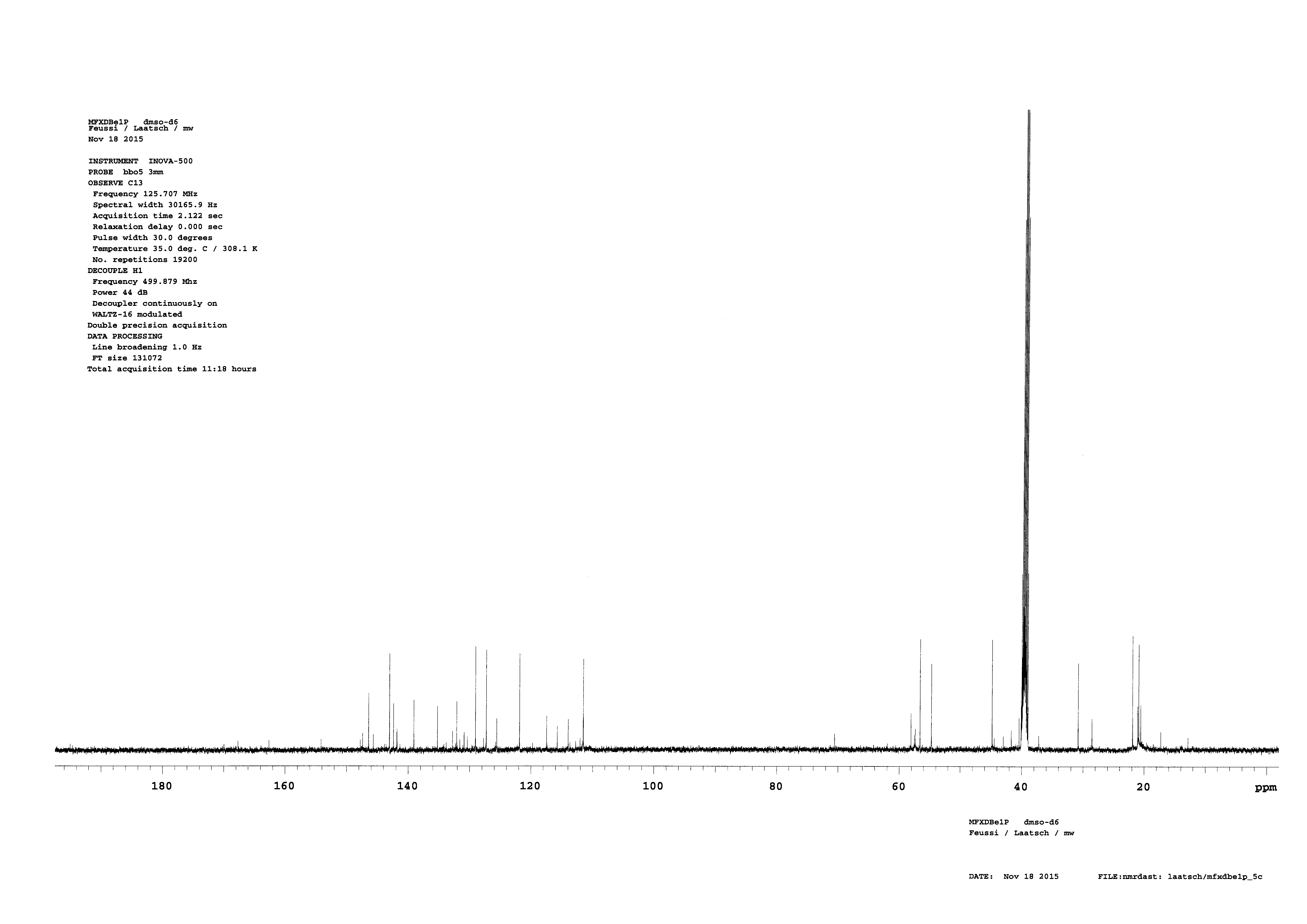


# Figure S11: ^13^C NMR spectrum (125 MHz, DMSO-*d*_6_) of 15-hydroxy-3,6-dihydrolactarazulene (2) and 15-hydroxy-6,7-dihydrolac­tarazulene (3)

# Figure S12: HSQC spectrum (300 MHz, DMSO-*d*_6_) of 15-hydroxy-3,6-dihydrolactarazulene (2) and 15-hydroxy-6,7-dihydrolactarazulene (3)

# Figure S13: Magnified HSQC spectrum (300 MHz, DMSO-*d*_6_) of 15-hydroxy-3,6-dihydrolactarazulene (2) and 15-hydroxy-6,7-dihydrolactarazulene (3)

# Figure S14: Magnified HSQC spectrum (300 MHz, DMSO-*d*_6_) of 15-hydroxy-3,6-dihydrolactarazulene (2) and 15-hydroxy-6,7-dihydrolac­tarazulene (3)

# Figure S15: Magnified HSQC spectrum (300 MHz, DMSO-*d*_6_) of 15-hydroxy-3,6-dihydrolactarazulene (2) and 15-hydroxy-6,7-dihydrolac­tarazulene (3)

**Figure S16**: HMBC spectrum (300 MHz, DMSO-*d*_6_) of 15-hydroxy-3,6-dihydrolactarazulene (**2**) and 15-hydroxy-6,7-dihydrolac­tarazulene (**3**)

# Figure S17: Magnified spectrum HMBC (300 MHz, DMSO-*d*_6_) of 15-hydroxy-3,6-dihydrolactarazulene (2) and 15-hydroxy-6,7-dihydrolac­tarazulene (3)

# Figure S18: Magnified HMBC spectrum (300 MHz, DMSO-*d*_6_) of 15-hydroxy-3,6-dihydrolactarazulene (2) and 15-hydroxy-6,7-dihydrolac­tarazulene (3)

# Figure S19: Magnified HMBC spectrum (300 MHz, DMSO-*d*_6_) of 15-hydroxy-3,6-dihydrolactarazulene (2) and 15-hydroxy-6,7-dihydrolac­tarazulene (3)

# Figure S20: Magnified HMBC spectrum (300 MHz, DMSO-*d*_6_) of 15-hydroxy-3,6-dihydrolactarazulene (2) and 15-hydroxy-6,7-dihydrolac­tarazulene (3)

# Figure S21: Magnified HMBC spectrum (300 MHz, DMSO-*d*_6_) of 15-hydroxy-3,6-dihydrolactarazulene (2) and 15-hydroxy-6,7-dihydrolac­tarazulene (3)

# Figure S22: COSY spectrum (300 MHz, DMSO-*d*_6_) of 15-hydroxy-3,6-dihydrolactarazulene (2) and 15-hydroxy-6,7-dihydrolac­tarazulene (3)

# Figure S23: Magnified COSY spectrum (300 MHz, DMSO-*d*_6_) of 15-hydroxy-3,6-dihydrolactarazulene (2) and 15-hydroxy-6,7-dihydrolac­tarazulene (3)

# Figure S24: Magnified COSY spectrum (300 MHz, DMSO-*d*_6_) of 15-hydroxy-3,6-dihydrolactarazulene (2) and 15-hydroxy-6,7-dihydrolac­tarazulene (3)

1. present address: Jilin University, School of Plant Science, Xian Road No. 5333, 130062 Changchun, Jilin, P.R. China [↑](#footnote-ref-2)
2. ^*^ Corresponding author: [hlaatsc@gwdg.de](mailto:hlaatsc@gwdg.de) [↑](#footnote-ref-3)
